# Supplementary material for: Do residential patterns affect women’s labor market performance? An empirical study based on CHFS data
Source: PLoS One. 2023 Nov 20;18(11):e0294558. doi: 10.1371/journal.pone.0294558 (PMC10659203; doi:10.1371/journal.pone.0294558)
Supplement: S1 Appendix — (DOCX) [file pone.0294558.s001.docx]

**S1 table 1. The CMP estimation results of the impact of residential patterns on female labor market participation (use *IV_1* and exclude the sample of families with migratory behavior).**

|  | **The national samples** | | |
| --- | --- | --- | --- |
|  | **first** | **second** | **margin** |
| IV_1 | 1.198^***^ |  |  |
|  | (8.477) |  |  |
| coresidence |  | 1.033^***^ | 0.311^***^ |
|  |  | (4.654) | (4.493) |
| age | 0.053^*^ | 0.123^***^ | 0.037^***^ |
|  | (1.710) | (4.038) | (4.097) |
| age_square | -0.094^**^ | -0.129^***^ | -0.039^***^ |
|  | (-2.410) | (-3.354) | (-3.396) |
| ethnic | -0.066 | -0.042 | -0.013 |
|  | (-1.100) | (-0.686) | (-0.687) |
| health | 0.048^**^ | -0.082^***^ | -0.025^***^ |
|  | (2.278) | (-4.089) | (-4.098) |
| edu | -0.026^**^ | 0.135^***^ | 0.041^***^ |
|  | (-2.064) | (10.963) | (11.504) |
| income_husband | -0.013^***^ | -0.015^***^ | -0.004^***^ |
|  | (-3.548) | (-3.473) | (-3.533) |
| children_number | 0.167^***^ | -0.121^***^ | -0.036^***^ |
|  | (4.630) | (-3.409) | (-3.391) |
| boy_number | -0.033 | 0.061 | 0.018 |
|  | (-0.812) | (1.606) | (1.607) |
| preschooler | 0.031 | -0.360^***^ | -0.108^***^ |
|  | (0.599) | (-6.955) | (-7.159) |
| adult_number | 0.554^***^ | -0.131 | -0.039 |
|  | (5.864) | (-1.331) | (-1.324) |
| _cons | -1.777^***^ | -2.197^***^ |  |
|  | (-2.807) | (-3.767) |  |
| atanhrho_12 | -0.598^***^ |  |  |
|  | (-2.897) |  |  |
| Observations | 6228 | 6228 | 6228 |
| Province FE | Yes | Yes | Yes |

Note: (1) *, **, and *** represent significance at the 10%, 5%, and 1% levels, respectively. (2) The numbers shown in parentheses are z values under robust standard errors. (3) The first and second stages shown in the table report the regression coefficients of the probit model, and the marginal coefficients report the average marginal effects of the second-stage probit model.

**S1 table 2. The impact of the number of parents living together on female labor market participation.**

|  | **The national samples** | | **The urban samples** | | **The rural samples** | |
| --- | --- | --- | --- | --- | --- | --- |
|  | **Probit** | **OLS** | **Probit** | **OLS** | **Probit** | **OLS** |
| coresidence_num | 0.037^***^ | 0.036^***^ | 0.044^***^ | 0.043^***^ | 0.008 | 0.006 |
|  | (4.105) | (4.283) | (3.853) | (4.137) | (0.581) | (0.416) |
| age | 0.042^***^ | 0.046^***^ | 0.051^***^ | 0.056^***^ | 0.029^**^ | 0.038^**^ |
|  | (4.803) | (4.732) | (4.892) | (4.697) | (2.044) | (2.291) |
| age_square | -0.047^***^ | -0.052^***^ | -0.061^***^ | -0.067^***^ | -0.029^*^ | -0.040^**^ |
|  | (-4.312) | (-4.286) | (-4.657) | (-4.461) | (-1.683) | (-2.006) |
| ethnic | -0.023 | -0.025 | 0.025 | 0.024 | -0.046^*^ | -0.048^*^ |
|  | (-1.251) | (-1.349) | (1.034) | (0.951) | (-1.664) | (-1.822) |
| health | -0.021^***^ | -0.022^***^ | -0.013^*^ | -0.015^*^ | -0.046^***^ | -0.046^***^ |
|  | (-3.417) | (-3.522) | (-1.709) | (-1.891) | (-4.611) | (-4.492) |
| edu | 0.041^***^ | 0.041^***^ | 0.054^***^ | 0.053^***^ | 0.028^***^ | 0.030^***^ |
|  | (11.882) | (12.290) | (13.736) | (13.615) | (2.829) | (3.055) |
| income_husband | -0.006^***^ | -0.006^***^ | -0.005^***^ | -0.005^***^ | -0.007^***^ | -0.007^***^ |
|  | (-5.544) | (-5.833) | (-3.476) | (-3.748) | (-3.625) | (-3.573) |
| children_number | -0.025^**^ | -0.027^**^ | -0.042^***^ | -0.046^***^ | -0.013 | -0.014 |
|  | (-2.420) | (-2.437) | (-3.110) | (-3.097) | (-0.837) | (-0.842) |
| boy_number | 0.019^*^ | 0.019^*^ | 0.026^*^ | 0.027^*^ | -0.010 | -0.009 |
|  | (1.670) | (1.673) | (1.912) | (1.949) | (-0.471) | (-0.423) |
| preschooler | -0.115^***^ | -0.118^***^ | -0.114^***^ | -0.113^***^ | -0.114^***^ | -0.127^***^ |
|  | (-7.807) | (-7.423) | (-6.544) | (-6.143) | (-4.375) | (-3.957) |
| adult_number | 0.012 | 0.012 | 0.058 | 0.057 | -0.035 | -0.041 |
|  | (0.462) | (0.431) | (1.416) | (1.551) | (-1.068) | (-1.115) |
| _cons |  | -0.207 |  | -0.475^**^ |  | -0.291 |
|  |  | (-1.057) |  | (-2.035) |  | (-0.679) |
| Observations | 6441 | 6441 | 4616 | 4616 | 1825 | 1825 |
| Province FE | Yes | Yes | Yes | Yes | Yes | Yes |
| Adj-R2 |  | 0.071 |  | 0.086 |  | 0.096 |

Note: (1) *, **, and *** represent significance at the 10%, 5%, and 1% levels, respectively. (2) The numbers shown in parentheses for the probit model are z values under robust standard errors, and the numbers shown in parentheses for the OLS model are t values under robust standard errors. (3) The probit model results report the average marginal effects.

**S1 table 3. The impact of the number of parents living together on female labor market income.**

|  | **The national samples** | | **The urban samples** | | **The rural samples** | |
| --- | --- | --- | --- | --- | --- | --- |
|  | **first** | **second** | **first** | **second** | **first** | **second** |
| coresidence_num | 0.123^***^ | 0.078^***^ | 0.145^***^ | 0.137^***^ | 0.031 | -0.007 |
|  | (4.091) | (2.677) | (3.836) | (4.027) | (0.581) | (-0.121) |
| age | 0.140^***^ | 0.162^***^ | 0.169^***^ | 0.212^***^ | 0.115^**^ | 0.059 |
|  | (4.782) | (4.350) | (4.860) | (4.804) | (2.034) | (0.612) |
| age_square | -0.157^***^ | -0.191^***^ | -0.202^***^ | -0.256^***^ | -0.116^*^ | -0.082 |
|  | (-4.297) | (-4.205) | (-4.629) | (-4.692) | (-1.677) | (-0.717) |
| ethnic | -0.077 | -0.043 | 0.082 | 0.017 | -0.181^*^ | 0.046 |
|  | (-1.250) | (-0.785) | (1.034) | (0.309) | (-1.657) | (0.330) |
| health | -0.071^***^ | -0.139^***^ | -0.043^*^ | -0.129^***^ | -0.182^***^ | -0.136^**^ |
|  | (-3.406) | (-6.604) | (-1.707) | (-5.913) | (-4.535) | (-2.030) |
| edu | 0.138^***^ | 0.341^***^ | 0.177^***^ | 0.391^***^ | 0.109^***^ | 0.151^***^ |
|  | (11.541) | (17.119) | (13.088) | (13.945) | (2.806) | (3.327) |
| income_husband | -0.020^***^ |  | -0.015^***^ |  | -0.027^***^ |  |
|  | (-5.504) |  | (-3.460) |  | (-3.597) |  |
| children_number | -0.085^**^ | -0.116^***^ | -0.139^***^ | -0.147^***^ | -0.053 | -0.112 |
|  | (-2.418) | (-3.147) | (-3.102) | (-3.203) | (-0.836) | (-1.502) |
| boy_number | 0.064^*^ | 0.016 | 0.085^*^ | 0.036 | -0.039 | 0.088 |
|  | (1.669) | (0.497) | (1.910) | (0.983) | (-0.471) | (0.918) |
| preschooler | -0.384^***^ | -0.302^***^ | -0.376^***^ | -0.366^***^ | -0.447^***^ | -0.122 |
|  | (-7.714) | (-4.427) | (-6.465) | (-5.187) | (-4.330) | (-0.691) |
| adult_number | 0.041 | 0.082 | 0.190 | 0.238^***^ | -0.136 | -0.105 |
|  | (0.462) | (0.934) | (1.415) | (2.839) | (-1.067) | (-0.469) |
| imr |  | 1.539^***^ |  | 1.973^***^ |  | 0.074 |
|  |  | (4.691) |  | (5.389) |  | (0.107) |
| _cons | -2.160^***^ | 6.079^***^ | -2.947^***^ | 4.733^***^ | -2.221^*^ | 9.612^***^ |
|  | (-3.670) | (7.247) | (-4.284) | (4.639) | (-1.683) | (3.986) |
| Observations | 6441 | 3044 | 4616 | 2534 | 1825 | 510 |
| Province FE | Yes | Yes | Yes | Yes | Yes | Yes |
| Pseudo R2 | 0.068 |  | 0.083 |  | 0.114 |  |
| Adj-R2 |  | 0.362 |  | 0.338 |  | 0.113 |

Note: (1) *, **, and *** represent significance at the 10%, 5%, and 1% levels, respectively. (2) The numbers shown in parentheses in the first stage are z values under robust standard errors, and the numbers shown in parentheses in the second stage are t values under robust standard errors. (3) The first stage shown in the table reports the regression coefficients of the probit model and not the average marginal effects.

**S1 table 4. The impact of residential patterns on male labor market participation.**

|  | **The national samples** | | **The urban samples** | | **The rural samples** | |
| --- | --- | --- | --- | --- | --- | --- |
|  | **Probit** | **OLS** | **Probit** | **OLS** | **Probit** | **OLS** |
| coresidence | -0.001 | -0.001 | -0.005 | -0.005 | -0.008 | -0.004 |
|  | (-0.157) | (-0.138) | (-0.538) | (-0.517) | (-0.745) | (-0.318) |
| age | 0.008^*^ | 0.018^***^ | 0.014^***^ | 0.031^***^ | -0.011 | -0.006 |
|  | (1.698) | (3.306) | (2.613) | (4.462) | (-1.128) | (-0.816) |
| age_square | -0.013^**^ | -0.025^***^ | -0.021^***^ | -0.043^***^ | 0.011 | 0.006 |
|  | (-2.444) | (-3.808) | (-3.450) | (-4.941) | (1.051) | (0.672) |
| ethnic | 0.014 | 0.019 | 0.026^**^ | 0.037^**^ | -0.001 | 0.005 |
|  | (1.434) | (1.600) | (2.077) | (2.153) | (-0.051) | (0.329) |
| health | -0.025^***^ | -0.030^***^ | -0.022^***^ | -0.029^***^ | -0.033^***^ | -0.036^***^ |
|  | (-6.954) | (-7.065) | (-5.128) | (-5.467) | (-5.498) | (-5.017) |
| edu | 0.008^***^ | 0.007^***^ | 0.011^***^ | 0.010^***^ | 0.010^*^ | 0.008^*^ |
|  | (3.731) | (3.723) | (4.750) | (4.675) | (1.835) | (1.882) |
| income_wife | -0.001^**^ | -0.002^**^ | -0.001 | -0.001 | -0.001 | -0.001 |
|  | (-2.230) | (-2.542) | (-1.063) | (-1.350) | (-0.909) | (-1.066) |
| children_number | -0.007 | -0.008 | -0.013^*^ | -0.015^*^ | -0.003 | -0.001 |
|  | (-1.244) | (-1.306) | (-1.754) | (-1.823) | (-0.311) | (-0.119) |
| boy_number | 0.003 | 0.002 | 0.002 | 0.001 | 0.009 | 0.006 |
|  | (0.493) | (0.376) | (0.256) | (0.156) | (0.820) | (0.465) |
| preschooler | -0.002 | -0.003 | 0.008 | 0.005 | -0.026^*^ | -0.028^*^ |
|  | (-0.246) | (-0.434) | (0.761) | (0.633) | (-1.824) | (-1.790) |
| adult_number | 0.019 | 0.019 | 0.014 | 0.014 | 0.027 | 0.017 |
|  | (1.164) | (1.095) | (0.747) | (0.489) | (1.257) | (1.212) |
| _cons |  | 0.653^***^ |  | 0.368^***^ |  | 1.227^***^ |
|  |  | (5.953) |  | (2.661) |  | (7.840) |
| Observations | 6455 | 6455 | 4618 | 4618 | 1778 | 1837 |
| Province FE | Yes | Yes | Yes | Yes | Yes | Yes |
| Adj-R2 |  | 0.038 |  | 0.057 |  | 0.028 |

Note: (1) *, **, and *** represent significance at the 10%, 5%, and 1% levels, respectively. (2) The numbers shown in parentheses for the probit model are z values under robust standard errors, and the numbers shown in parentheses for the OLS model are t values under robust standard errors. (3) The probit model results report the average marginal effects.

**S1 table 5. The impact of residential patterns on male labor market income.**

|  | **The national samples** | | **The urban samples** | | **The rural samples** | |
| --- | --- | --- | --- | --- | --- | --- |
|  | **first** | **second** | **first** | **second** | **first** | **second** |
| coresidence | -0.010 | -0.080^**^ | -0.043 | -0.057 | -0.090 | -0.080 |
|  | (-0.157) | (-2.399) | (-0.539) | (-1.461) | (-0.749) | (-1.206) |
| age | 0.071^*^ | 0.060^**^ | 0.123^***^ | 0.037 | -0.112 | 0.011 |
|  | (1.698) | (2.398) | (2.607) | (1.201) | (-1.133) | (0.198) |
| age_square | -0.115^**^ | -0.088^***^ | -0.184^***^ | -0.053 | 0.114 | -0.043 |
|  | (-2.445) | (-2.808) | (-3.443) | (-1.351) | (1.055) | (-0.653) |
| ethnic | 0.121 | 0.187^***^ | 0.228^**^ | 0.056 | -0.008 | 0.211^**^ |
|  | (1.433) | (3.520) | (2.076) | (0.916) | (-0.051) | (2.149) |
| health | -0.220^***^ | -0.140^***^ | -0.194^***^ | -0.098^***^ | -0.352^***^ | -0.176^***^ |
|  | (-6.937) | (-4.690) | (-5.098) | (-3.690) | (-5.661) | (-2.838) |
| edu | 0.068^***^ | 0.242^***^ | 0.097^***^ | 0.220^***^ | 0.106^*^ | 0.182^***^ |
|  | (3.750) | (26.281) | (4.789) | (19.614) | (1.857) | (6.133) |
| income_wife | -0.013^**^ |  | -0.007 |  | -0.011 |  |
|  | (-2.226) |  | (-1.061) |  | (-0.909) |  |
| children_number | -0.063 | -0.070^**^ | -0.112^*^ | -0.009 | -0.030 | -0.087^*^ |
|  | (-1.244) | (-2.435) | (-1.755) | (-0.238) | (-0.311) | (-1.698) |
| boy_number | 0.028 | 0.039 | 0.017 | 0.037 | 0.099 | 0.073 |
|  | (0.493) | (1.368) | (0.256) | (1.234) | (0.821) | (1.075) |
| preschooler | -0.019 | 0.074^**^ | 0.067 | 0.054 | -0.274^*^ | -0.019 |
|  | (-0.246) | (1.978) | (0.761) | (1.314) | (-1.833) | (-0.179) |
| adult_number | 0.167 | 0.082 | 0.123 | 0.015 | 0.283 | 0.145 |
|  | (1.165) | (1.183) | (0.747) | (0.165) | (1.267) | (1.351) |
| imr |  | 1.355^**^ |  | 0.661 |  | 1.340 |
|  |  | (2.410) |  | (1.473) |  | (1.384) |
| _cons | 0.787 | 9.363^***^ | -0.477 | 9.899^***^ | 5.497^**^ | 9.525^***^ |
|  | (0.853) | (18.260) | (-0.463) | (15.702) | (2.470) | (7.438) |
| Observations | 6455 | 4296 | 4618 | 3288 | 1778 | 963 |
| Province FE | Yes | Yes | Yes | Yes | Yes | Yes |
| Pseudo R2 | 0.085 |  | 0.115 |  | 0.116 |  |
| Adj-R2 |  | 0.347 |  | 0.303 |  | 0.233 |

Note: (1) *, **, and *** represent significance at the 10%, 5%, and 1% levels, respectively. (2) The numbers shown in parentheses in the first stage are z values under robust standard errors, and the numbers shown in parentheses in the second stage are t values under robust standard errors. (3) The first stage shown in the table reports the regression coefficients of the probit model and not the average marginal effects.

**S1 table 6. The impact of residential patterns on female labor market participation (city-level fixed effects).**

|  | **The national samples** | | **The urban samples** | | **The rural samples** | |
| --- | --- | --- | --- | --- | --- | --- |
|  | **Probit** | **OLS** | **Probit** | **OLS** | **Probit** | **OLS** |
| coresidence | 0.043^***^ | 0.043^***^ | 0.054^***^ | 0.054^***^ | 0.009 | 0.006 |
|  | (3.283) | (3.281) | (3.162) | (3.199) | (0.371) | (0.268) |
| age | 0.039^***^ | 0.044^***^ | 0.049^***^ | 0.053^***^ | 0.032^*^ | 0.036^**^ |
|  | (4.628) | (4.512) | (4.613) | (4.380) | (1.818) | (2.063) |
| age_square | -0.044^***^ | -0.050^***^ | -0.058^***^ | -0.063^***^ | -0.031 | -0.037^*^ |
|  | (-4.152) | (-4.087) | (-4.405) | (-4.163) | (-1.424) | (-1.774) |
| ethnic | -0.026 | -0.033 | 0.005 | -0.002 | -0.047 | -0.048 |
|  | (-1.207) | (-1.457) | (0.176) | (-0.065) | (-1.030) | (-1.214) |
| health | -0.025^***^ | -0.025^***^ | -0.015^*^ | -0.016^**^ | -0.059^***^ | -0.048^***^ |
|  | (-4.093) | (-3.943) | (-1.956) | (-1.974) | (-4.938) | (-4.428) |
| edu | 0.044^***^ | 0.045^***^ | 0.055^***^ | 0.055^***^ | 0.022^*^ | 0.021^*^ |
|  | (12.181) | (12.136) | (13.266) | (12.696) | (1.699) | (1.774) |
| income_husband | -0.005^***^ | -0.005^***^ | -0.004^***^ | -0.004^***^ | -0.006^***^ | -0.005^**^ |
|  | (-4.880) | (-4.857) | (-3.224) | (-3.226) | (-2.694) | (-2.426) |
| children_number | -0.034^***^ | -0.035^***^ | -0.051^***^ | -0.052^***^ | -0.001 | -0.001 |
|  | (-3.213) | (-3.054) | (-3.650) | (-3.461) | (-0.059) | (-0.046) |
| boy_number | 0.018 | 0.019^*^ | 0.023^*^ | 0.024^*^ | -0.015 | -0.010 |
|  | (1.598) | (1.655) | (1.719) | (1.750) | (-0.600) | (-0.447) |
| preschooler | -0.112^***^ | -0.115^***^ | -0.114^***^ | -0.112^***^ | -0.138^***^ | -0.132^***^ |
|  | (-7.671) | (-7.179) | (-6.469) | (-5.904) | (-4.351) | (-3.792) |
| adult_number | 0.008 | 0.009 | 0.056 | 0.052 | -0.043 | -0.036 |
|  | (0.324) | (0.334) | (1.395) | (1.466) | (-1.090) | (-0.920) |
| _cons |  | -0.406 |  | -0.984^***^ |  | -0.209 |
|  |  | (-0.994) |  | (-3.573) |  | (-0.388) |
| Observations | 6388 | 6441 | 4525 | 4616 | 1434 | 1825 |
| Province FE | Yes | Yes | Yes | Yes | Yes | Yes |
| Adj-R2 |  | 0.092 |  | 0.106 |  | 0.105 |

Note: (1) *, **, and *** represent significance at the 10%, 5%, and 1% levels, respectively. (2) The numbers shown in parentheses for the probit model are z values under robust standard errors, and the numbers shown in parentheses for the OLS model are t values under robust standard errors. (3) The probit model results report the average marginal effects.

**S1 table 7. The impact of residential patterns on female labor market income (city-level fixed effects).**

|  | **The national samples** | | **The urban samples** | | **The rural samples** | |
| --- | --- | --- | --- | --- | --- | --- |
|  | **first** | **second** | **first** | **second** | **first** | **second** |
| coresidence | 0.152^***^ | 0.092^**^ | 0.188^***^ | 0.118^**^ | 0.033 | -0.103 |
|  | (3.277) | (2.045) | (3.151) | (2.378) | (0.371) | (-0.652) |
| age | 0.139^***^ | 0.142^***^ | 0.168^***^ | 0.127^***^ | 0.117^*^ | -0.030 |
|  | (4.608) | (3.740) | (4.584) | (2.927) | (1.809) | (-0.193) |
| age_square | -0.156^***^ | -0.167^***^ | -0.202^***^ | -0.155^***^ | -0.112 | 0.016 |
|  | (-4.138) | (-3.565) | (-4.380) | (-2.852) | (-1.419) | (0.092) |
| ethnic | -0.093 | -0.120^**^ | 0.016 | -0.038 | -0.172 | -0.123 |
|  | (-1.206) | (-1.966) | (0.176) | (-0.611) | (-1.029) | (-0.439) |
| health | -0.088^***^ | -0.125^***^ | -0.052^*^ | -0.097^***^ | -0.215^***^ | 0.001 |
|  | (-4.079) | (-5.941) | (-1.955) | (-4.474) | (-4.828) | (0.007) |
| edu | 0.157^***^ | 0.316^***^ | 0.191^***^ | 0.307^***^ | 0.080^*^ | 0.129^*^ |
|  | (11.812) | (16.420) | (12.670) | (12.420) | (1.691) | (1.868) |
| income_husband | -0.019^***^ |  | -0.015^***^ |  | -0.023^***^ |  |
|  | (-4.858) |  | (-3.212) |  | (-2.683) |  |
| children_number | -0.122^***^ | -0.092^**^ | -0.176^***^ | -0.073 | -0.004 | -0.074 |
|  | (-3.208) | (-2.232) | (-3.638) | (-1.465) | (-0.059) | (-0.613) |
| boy_number | 0.064 | 0.028 | 0.081^*^ | 0.012 | -0.055 | 0.079 |
|  | (1.598) | (0.830) | (1.718) | (0.324) | (-0.600) | (0.579) |
| preschooler | -0.395^***^ | -0.286^***^ | -0.395^***^ | -0.243^***^ | -0.500^***^ | 0.394 |
|  | (-7.581) | (-4.769) | (-6.394) | (-3.800) | (-4.293) | (1.285) |
| adult_number | 0.029 | 0.082 | 0.193 | 0.109 | -0.157 | 0.092 |
|  | (0.324) | (0.920) | (1.395) | (1.177) | (-1.089) | (0.287) |
| imr |  | 1.356^***^ |  | 1.049^***^ |  | -0.983 |
|  |  | (4.985) |  | (3.570) |  | (-1.019) |
| _cons | -2.850^***^ | 4.929^***^ | -4.694^***^ | 5.413^***^ | -2.283 | 10.389^***^ |
|  | (-2.680) | (5.016) | (-5.086) | (4.671) | (-1.418) | (2.850) |
| Observations | 6388 | 3029 | 4525 | 2493 | 1434 | 379 |
| Province FE | Yes | Yes | Yes | Yes | Yes | Yes |
| Pseudo R2 | 0.116 |  | 0.133 |  | 0.151 |  |
| Adj-R2 |  | 0.389 |  | 0.365 |  | 0.099 |

Note: (1) *, **, and *** represent significance at the 10%, 5%, and 1% levels, respectively. (2) The numbers shown in parentheses in the first stage are z values under robust standard errors, and the numbers shown in parentheses in the second stage are t values under robust standard errors. (3) The first stage shown in the table reports the regression coefficients of the probit model and not the average marginal effects.
