# Supplementary material for: Do residential patterns affect women’s labor market performance? An empirical study based on CHFS data
Source: PLoS One. 2023 Nov 20;18(11):e0294558. doi: 10.1371/journal.pone.0294558 (PMC10659203; doi:10.1371/journal.pone.0294558)
Supplement: Attachment — Submitted filename: Response to Reviewers.docx [file pone.0294558.s003.docx]

# Response to Review Comments

# (Manuscript Number: PONE-D-22-34476R1)

Dear Editor,

We would like to express our gratitude to you and the reviewers for investing your valuable time in reviewing our paper and for providing insightful comments. It was these insightful remarks that allowed us to make potential improvements in the current version. The authors have taken great care to consider each comment and have made every effort to address them. We hope that the revised manuscript now meets your high standards. We would welcome any further constructive comments. Below, we have provided point-by-point responses, with all modifications in the manuscript highlighted in yellow.

**Response to the reviewers’ comments:**

**Comment 1:** Research published in PLOS ONE must have been conducted to the highest ethical standards. Because your study does involve survey-based research, you must provide evidence that you have received approval from the relevant responsible body. If approval was not obtained, you must explain why it was not required (for further details, see the criteria for publication guidelines at <https://journals.plos.org/plosone/s/criteria-for-publication>).

**Response:** The data utilized in this paper is derived from the China Household Financial Survey (CHFS), which is managed and organized by the Survey and Research Center for China Household Finance at the Southwestern University of Finance and Economics. The CHFS is a nationwide sample survey designed to gather relevant information about household finance at the micro level, offering a comprehensive and detailed depiction of household economic and financial behaviors. The publicly available data from 2011 to 2019 can be accessed by the public through a designated website for academic research, free of charge. The survey was conducted with the informed consent of each household member. In the publicly available data, personal information is anonymized, and each family and individual is assigned a unique identifier. Consequently, the survey does not involve human experimentation and does not disclose any private information about individuals or families. This paper exclusively employs the data from this research for academic research and non-commercial purposes. As such, ethical approval is not required.

**Comment 2:** The other requirement is a kind suggestion to add the keyword "female labor" to the appropriate entries and to change the keyword "labor market participation" for “labor market inclusion.”

**Response:** Thank you for your suggestion. We have included "female labor" in the list of keywords and made the change from "labor market participation" to "labor market inclusion," as highlighted in yellow in the manuscript.

**Comment 3:** Please review your reference list to ensure that it is complete and correct. If you have cited papers that have been retracted, please include the rationale for doing so in the manuscript text, or remove these references and replace them with relevant current references. Any changes to the reference list should be mentioned in the rebuttal letter that accompanies your revised manuscript. If you need to cite a retracted article, indicate the article’s retracted status in the References list and also include a citation and full reference for the retraction notice.
**Response:** Thank you for your advice. We have thoroughly reviewed the references once more and made the following improvements: ensuring that page numbers for relevant documents are complete, correcting any spelling errors, and carefully proofreading author names. The altered references have been marked with yellow highlighting. Upon this review, we have confirmed that we did not cite any literature that has been retracted.
